# Systematic review of measurement properties of the Canadian Occupational Performance Measure in geriatric rehabilitation

**DOI:** 10.1007/s41999-022-00692-8

**Published:** 2022-08-23

**Authors:** Margot W. M. de Waal, Miriam L. Haaksma, Arno J. Doornebosch, Rimmie Meijs, Wilco P. Achterberg

**Affiliations:** 1 University Network for the Care Sector Zuid-Holland, Leiden University Medical Center, Leiden, The Netherlands; 2 Department of Public Health and Primary Care, Leiden University Medical Center, Leiden, The Netherlands; 3 Topaz Revitel, Leiden, The Netherlands

**Keywords:** COPM, Geriatric rehabilitation, Aged, Validity, Responsiveness, Reliability

## Abstract

**Purpose:**

The Canadian Occupational Performance Measure (COPM) is used to inventory problems experienced by the patient to set goals and evaluate treatment. We aimed to make a systematic overview of measurement properties for people in geriatric rehabilitation.

**Methods:**

Seven electronic databases were searched for psychometric studies investigating content validity, construct validity, responsiveness, or reliability of the COPM in geriatric rehabilitation populations aged ≥ 60 years. Two reviewers independently abstracted data and assessed methodological quality from included studies.

**Results:**

Of 292 identified articles, 13 studies were included. The COPM showed good test–retest reliability (two studies), moderate inter-rater reliability (one study), and good content validity (one study with some risk of bias). Four studies with minimal risk of bias showed good construct validity as their hypotheses were confirmed. Responsiveness was moderate in three studies with adequate methodological quality.

**Conclusion:**

All measurement properties have been studied in geriatric rehabilitation populations, and indicate that the COPM gives relevant information for geriatric rehabilitation, and scores can be assessed reliably and are responsive to change. Although there were many studies on construct validity, authors had different opinions on what exactly COPM-scores tell us, as they used a variety of comparator instruments and different hypotheses. Consensus on exact interpretation of the scores is needed.

**Supplementary Information:**

The online version contains supplementary material available at 10.1007/s41999-022-00692-8.

## Key summary points

**Aim:**

To make a systematic overview of measurement properties of the Canadian Occupational Performance Measure (COPM) for people in geriatric rehabilitation.

**Findings:**

COPM showed moderate inter-rater reliability, good test–retest reliability, good content and construct validity, and moderate responsiveness in geriatric rehabilitation. When studying construct validity, authors used a variety of comparator instruments and different hypotheses.

**Message:**

This overview of properties shows that the COPM gives relevant information for geriatric rehabilitation, and scores can be assessed reliably and are responsive to change.

**Supplementary Information:**

The online version contains supplementary material available at 10.1007/s41999-022-00692-8.

## Introduction

In our aging society, a growing number of older people experience a sudden decline in functioning, for instance due to hip fracture or stroke. As a consequence, they lose their ability to live independently, and participation in society is limited. Geriatric rehabilitation is rehabilitation for persons with multimorbidity and frailty, that offers treatment focusing on improving functioning and participation. Geriatric rehabilitation is offered in widely different settings across the world, depending on national reimbursement policies and local availability [1]. It can be community-based or hospital-based, provided in skilled nursing facilities or on an outpatient basis [2].

During geriatric rehabilitation, it is important to agree on individual goals that both individual patients and professionals regard as important. The Canadian Occupational Performance Measure (COPM) can be useful to explore problems in daily functioning [3]. It is a personalized, client-centered instrument. In a semi-structured interview, the care professional explores occupational performance problems experienced by the patient in three areas of everyday living: self-care, productivity, and leisure. Patients are asked to select up to five of the most important problems and rate their own level of performance and satisfaction with performance on a 10-point scale. From the list of most important problems, two average scores are calculated: the COPM-Performance score (COPM-P) and COPM-Satisfaction score (COPM-S), that can range from 1 to 10. The COPM has been shown to give relevant and comprehensive information, i.e., good content validity, in various populations [4–6]. As such, it can provide valuable input for decisions on intervention goals and to guide the rehabilitation process.

Currently, the COPM is mostly used in adult rehabilitation and slowly finds its way in geriatric rehabilitation. It is important to understand the value of (changes in) COPM-scores, and whether they are helpful in evaluating treatment in this setting. Although there are some reviews on measurement properties of the COPM in a broad range of populations, there are none specifically for the geriatric rehabilitation population [7, 8]. Patients in this setting have specific characteristics, such as older age, comorbidity, frailty, and cognitive impairment that may influence the usability, reliability, and validity of the COPM [2]. We, therefore, performed a systematic review to examine whether the COPM is a valid, reliable, and responsive instrument for measuring problems in occupational performance in the geriatric rehabilitation setting .

## Methods

The following seven databases were searched on 28th of March 2019 and updated on 14th of March 2022: PubMed, Embase, Emcare, Web of Science, Cochrane Library, Academic Search Premier, and PsychINFO. The search string included the terms ‘Canadian occupational performance’ and one of the following ‘Aged, elderly, geriatric, homes for the Aged, Health services for the Aged, Senior Centers, old/older/aging [population or person]’ (see supplement 1 for full search string).

To select publications, we used the following eligibility criteria: (1) COPM is mentioned as an assessment tool in title or abstract; (2) the publication reports on measurement properties of interest: content validity (with interviews; exclusion of studies solely describing the total and prioritized problems), construct validity, reliability, and responsiveness; (3) date of publication from 1991 onwards; (4) mean or median age of population 60 years and over; (5) studies in rehabilitation setting.

For the assessment of the publications, we used the method and checklists described in the COSMIN manual for systematic reviews of PROMS [9, 10]. For each of the evaluated measurement properties, the methodological quality was evaluated using the criteria for risk of bias outlined in the respective boxes of the COSMIN manual. Each box contains a checklist, and the final methodological quality is determined using the ‘worst score counts’ method. This is a rather stringent method, and we will report the reason for the worst count. One author (MdW) screened all publications. Two authors (MdW and MH) independently extracted the information from selected publications, and then compared their information. When in doubt, co-authors were consulted (AD, WA). For the syntheses, we grouped the studies for each measurement property:

- Content validity is the degree to which the content of an instrument is an adequate reflection of the construct to be measured. We gathered information on three aspects: (1) relevance for construct, for target population, and for context of use; (2) comprehensiveness; and (3) comprehensibility [11].
- Construct validity is the degree to which the COPM-scores are consistent with hypotheses. We gathered information on method used, intervention and follow-up time, and results of analyses: correlations of scores between COPM and comparator instruments (convergent or divergent validity), and differences in scores between groups (discriminative validity). Reports on predictive validity were excluded. We described whether authors defined hypotheses for expected correlations beforehand. We also described the size of the correlations regardless of statistical significance, in accordance with COSMIN. In reporting, we interpreted correlations of sizes < 0.30 as low, 0.30–0.50 as moderate, and ≥ 0.50 as high [12].
- Responsiveness is the ability to detect change over time in the construct to be measured. This is done by comparing changing scores on the COPM to scores on other instruments, or by looking at differences between subgroups, or by comparing change before and after intervention. We gathered information on method used, results of analyses, and hypotheses formulated by authors regarding the effect of the applied intervention. To assess whether change can be expected, we gathered information on the intervention and follow-up time.
- Reliability is the degree to which the measurement is free from measurement error, over time (test–retest reliability) or between persons (intra-rater or inter-rater reliability). We gathered information on correlations or ICC.

## Results

### Characteristics of included studies (*n* = 13)

**Fig. 1:**
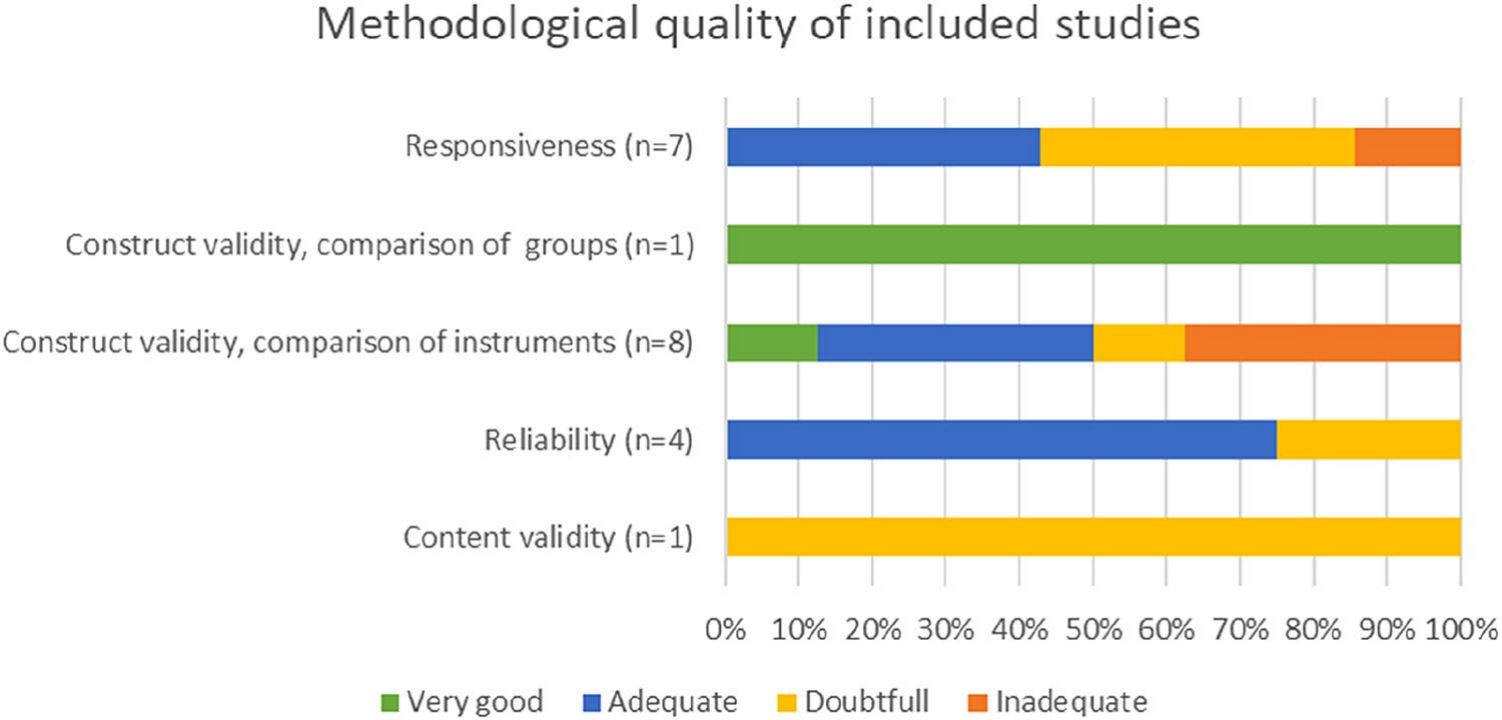
Methodological quality, reported as the lowest score of all items from the COSMIN checklist, for each measurement property of the 13 included studies

The literature search resulted in 292 publications, of which 43 reported on measurement properties including clinical utility. After reading full texts, 12 publications reporting on 13 studies were selected to be included (see supplement 2 figure S2.1, PRISMA flow diagram). They reported on content validity one time, construct validity eight times, responsiveness seven times, and reliability three times (see Table 1). Almost two-thirds of the studies (eight of 13) included patients with various diagnoses. The other studies included patients with one type of diagnosis, i.e., stroke, hip fractures, rheumatic diseases, COPD, and Parkinson’s disease, respectively. The settings were described as acute (one in Canada) or sub-acute in-hospital rehabilitation (two studies in Australia), outpatient setting (one study in Norway, one in the Netherlands, and one in the UK), community- or home-based rehabilitation (one in Norway, one in the Netherlands), or a combination of settings (four studies in Denmark, one in Sweden). Baseline mean or median COPM-Performance (COPM-P) scores ranged from 2.2 to 5.2. The baseline mean or median COPM-Satisfaction (COPM-S) scores ranged from 2.7 to 7.4 (outlier). Mean COPM-scores for Performance and Satisfaction differed only slightly, except for two studies, one with lower COPM-P scores [13] and one with much higher COPM-P scores [14] compared to the COPM-S scores. Three studies did not report baseline COPM-scores. Methodological quality was rated for each measurement property. In the one study on content validity and one study on reliability, the methodological quality was rated as doubtful. For construct validity and responsiveness, methodological quality was rated as very good to adequate in half of the studies (see Fig. 1).

**Table 1:** Characteristics of studies included in literature review (13 studies in 12 publications)

Author	Setting	Population—patients	Age and sex of patients	Measures, reported in this paper	COPM-P baseline	COPM-S baseline
Cup et al. [16]	The Netherlands, stroke services, hospital outpatients	Stroke patients (*n* = 26), with residual impairments and disabilities; living at home (54%) in nursing home (35%) or rehabilitation center (11%)	Mean 68 (SD 15); 58% women	Construct validity reliability (test–retest)	Mean 3.5 (SD 1.8)	Mean 3.3 (SD 1.9)
Edwards et al. [19]	Canada, hospital admission for surgery	Patients with hip fracture (*n* = 50), aged 65 +	Mean 80.8 (SD 7.3); 76% women	Construct validity, responsiveness	Mean 2.2 (SD 1.5)	Mean 2.7 (SD 2.1)
Enemark Larsen [13]	Denmark, two settings, a regional hospital and a community-based rehabilitation center	Patients (*n* = 109), aged 18+, in need for rehabilitation due to hand/knee surgery, orthopedic, medical or neurologic diseases or injuries	Mean 64.7, range 16–96; 56% women	Construct validity	Mean 3.6 (SD 1.7)	Mean 2.7 (SD 1.7)
Enemark Larsen [23] study A (2R)	Denmark, regional hospital and community rehabilitation center with in- and outpatients	Patients (*n* = 83) with hand- or knee surgery; with orthopedic, medical or neurological diseases or injuries	Mean 63.9 (SD 16.2); 55% women	Interrater reliability	Mean 3.7 (SD 1.5)	Mean 2.8 (SD 1.5)
Enemark Larsen [23] study B (1R)	Denmark, regional hospitals and communication rehabilitation center with outpatients	Patients (*n* = 68) with hand- or knee surgery 5 years ago; with orthopedic, medical or neurological diseases or injuries	Mean 70.7 (SD 10.7); 60% women	Test–retest reliability	Mean 5.2 (SD 2.3)	Mean 5.0 (SD 2.6)
Enemark Larsen [20]	Denmark, regional hospital and two community-based rehabilitation centers with in- and outpatients	Patients (*n* = 88), aged 18+, in need for rehabilitation due to hand/knee surgery, orthopedic, medical or neurological diseases or injuries	Mean 64.5 (SD 15.4) range 16–90; 55% women	Responsiveness, minimal important change (MIC)	NA	NA
Kjeken [22]	Norway, rheumatology outpatients	Patients (*n* = 79) with hand osteoarthritis, aged 50–70 (responsiveness *n* = 65)	Mean 63.2 (SD 5.4); 95% women	Construct validity, responsiveness	NA	NA
Poerbodipoero [15]	The Netherlands, community-dwelling participants in RCT on home-based OT	Patients with Parkinson’s disease (*n* = 109), aged 18+, with MMSE ≥ 24	Mean 68.7 (SD 9.7); 38% women	Construct validity (COPM as comparator)	Median 4.3 (range 2.0–6.7)	Median 4.2 (range 1.4–7.5)
Roe [14]	Australia, sub-acute rehabilitation in a general hospital	Patients (*n* = 50) aged 65 and over, with various diagnoses or disabilities, without cognitive impairment	Mean 78.2 (SD 7.2); 64% women	Construct validity, responsiveness	Mean 3.8 (SD 2.3)	Mean 7.4 (SD 1.9)
Sewell L [24]	United Kingdom, outpatient-based pulmonary rehabilitation program	Patients with COPD (*n* = 15), clinically stable	Mean 67.1 (SD 7.4); 47% women	Reliability (test–retest)	Mean 4.3 (SD 1.6)	Mean 3.7 (SD 1.8)
Thyer [18]	Australia, sub-acute rehabilitation in large health service	Patients (*n* = 36), aged 18+, with various diagnoses, stable and without cognitive impairment, non-vulnerable	Mean 75.4 (SD 11.6), range 41–93; 56% women	Construct validity	Mean 3.7 (SD 1.9)	Mean 3.7 (SD 2.1)
Tuntland [6]	Norway, community-dwelling participants in controlled trial on multidisciplinary home-based rehabilitation (reablement)	Home-dwelling older adults (*n* = 225), aged 65+, with various health conditions, enrolled in a trial	Mean 80.8 (SD 6.7), range 65–95; 72% women	Content validity, construct validity, responsiveness, minimal important change (MIC)	Mean 3.5 (SD 1.7)	Mean 3.4 (SD 1.7)
Wressle [21]	Sweden, inpatients in geriatric or neurological wards and outpatients in neurological day-care	Patients with neurologic or orthopedic or other diagnoses (*n* = 108)	Median 78 (22–93); 67% women	Responsiveness	NA	NA

*NA* not available, *OT* occupational therapist, *RCT* randomized controlled trial, *SD* standard deviation, *MIC* minimal important change

### Content validity

Content validity was reported in one study [6]; see supplement 3 (table S3.1). In this study, the constructs of COPM were described as occupational performance and satisfaction with performance. Patients first finished the COPM-interview, and in addition were asked about coverage of occupations and experiences. Next, these answers and COPM-data were used to answer predefined assumptions on content validity. Tuntland et al. concluded that the COPM showed good content validity on the topics relevance, comprehensiveness, and comprehensibility.

For the topic comprehensibility, methodological quality was rated as adequate. For the topics relevance and comprehensiveness of the COPM-items, methodological quality was rated as doubtful due to the item ‘analysis by 1 instead of 2 researchers’, as the involvement of a second researcher was not explicitly described in the text. All other items on appropriate of methods were rated as very good or adequate.

### Construct validity

Construct validity was reported in eight studies; see Table 2. All studies analyzed construct validity by comparing scores between instruments. One study [15] also analyzed discriminative construct validity by comparing groups, as presented in a separate row in Table 2. All but one study [16] analyzed both COPM-P and COPM-S scores. Scores were compared with 11 other instruments for functioning, and for example with instruments for quality of life (EQ-5D, WHO-5), mental functioning (SF-36 mental, FIM cognitive), impact of sickness (AIMS2, SA-SIP30), or coping (SOC-13). One instrument shows many similarities with the COPM: the OSA ‘Occupational Self-Assessment’, with competences, values, and priorities for change [13, 17], although it has a predefined list of competences.

**Table 2:** Summary of findings on construct validity (*n* = 8 studies)

Author	Construct validity, method used	Comparators functioning	Comparators other	Results correlations COPM-P	Results correlations COPM-S	Hypotheses of authors	Hypotheses confirmed?	Remarks	Methodologic quality (item with lowest score)
Cup et al. [16]	Correlations between COPM-P scores and comparators; using Spearman’s rho	BI, FAI, Rankin Scale	SA-SIP30, EQ-5D	Low with BI, FAI, Rankin, SA-SIP30, EQ-5D		Hypothesized that COPM-performance scores are unrelated to scores on frequently used standardized functional measures (called ‘discriminant validity’)	Yes, low correlations	Quote: ‘The COPM evaluates patient-unique problems that are not evaluated by frequently used standardized functional measures.’	Inadequate (properties other instruments are not reported)
Edwards et al. [19]	Correlations between COPM-scores and comparator; using Pearson’s correlation coefficient; at two time points, T1 = baseline 3–5d after surgery, T4 = 3 months after discharge from rehabilitation	WOMAC function		Moderate (T1) to high (T4) with WOMAC function	Moderate (T1) to high (T4) with WOMAC function	Hypothesized that results from measures designed for a similar purpose would correlate	Yes, correlations	No hypotheses on size of correlations	Adequate (properties WOMAC for present population not reported)
Enemark Larsen [13]	Correlations between COPM-scores and comparators; using Spearman’s rho	OSA competence and values	EQ-5D-5L, WHO-5	Low with OSA-C, EQ-5D-5L vas mobility self-care; moderate (only) with EQ-5D-5L activities	Low with OSA-V, WHO-5, EQ-5D-5L vas pain anxiety	Hypothesized moderate correlation with OSA-C and OSA-V, and low correlations with WHO-5 and relevant items and vas of EQ-5D-5L	Partly; low correlations with EQ-5D/WHO-5, no moderate correlations with OSA	Quote: ‘This supports the notion that COPM provides information that is not obtainable with predefined items in the other standardized measures, as they measure an altogether different construct’	Inadequate (properties other instruments are not reported)
Kjeken [22]	Correlations of scores at baseline between COPM and comparators (self-reported); using Pearson’s correlation coefficient	MHAQ, AUSCAN function, WOMAC function, disease activity		Low with MHAQ, AUSCAN function, WOMAC function; moderate with disease activity	Low with MHAQ, AUSCAN function, WOMAC function; moderate with disease activity	Hypothesized a low (0–0.3) to moderate (0.3–0.7) correlation with the self-reported health status measures; and disease activity is one factor determining (satisfaction with) occupational performance	Yes, low-to-moderate correlations	Quote: ‘No higher correlations expected, as COPM is constructed as an individual measure and other instruments measure functions that are important at group level’	Inadequate (properties other instruments are not reported)
Poerbodi-poero [15]	Correlations between COPM-scores and comparator; using Spearman’s rho	ACS-NL		Weak correlations with ACS-performance	Weak correlations with ACS-satisfaction	Hypothesized moderate correlations (0.3–0.6) between ACS-P and COPM-P, and between ACS-S and COPM-S	No, no moderate correlations	Study aimed to assess the construct validity of the ACS-NL, with use of the COPM as a comparator instrument. COPM is thus viewed as a valid comparator measure in this study	Doubtful (properties comparator unknown, unclear number excluded)
Poerbodi-poero [15]	COPM-scores are compared between groups with mild and moderate PD severity; using the Mann–Whitney *U* test	N/A (discriminative validity)		COPM-P differs between mild and moderate Parkinson’s’ disease	COPM-S differs between mild and moderate Parkinson’s’ disease	Hypothesized that there will be a difference between groups (mild-moderate) as moderate PD severity can severely impact activities	Yes, differences between groups		Very good
Roe [14]	Correlation of scores at discharge between COPM and comparators; using Spearman’s rho with bootstrapping	FIM total, FIM motor, SF-36 physical	FIM cognitive, SF-36 mental	Low with FIM total, FIM motor, SF-36 physical, SF-36 mental	Moderate with FIM total, FIM motor, SF-36 physical; low with FIM cognitive, SF-36 mental	Hypotheses that scores were expected to be ‘significantly correlated’; no hypotheses on size of correlations	Yes, correlations ‘significant’	No hypotheses on size of correlations. Unlike other studies, high baseline COPM-S scores compared to COPM-P scores	Adequate (SF-36 some properties reported but not sure if these apply to study population)
Thyer [18]	Correlation of scores at admission between COPM and comparators; using Pearson’s *r* with bootstrapping	FIM total, FIM motor, SF-36 physical	FIM cognitive, SF-36 mental	Moderate with FIM total, FIM physical; low with FIM cognitive, SF-36 physical, SF-36 mental (at admission)	Moderate with FIM total, FIM physical; low with FIM cognitive, SF-36 physical, SF-36 mental (at admission)	None mentioned	N/A	No hypotheses on size of correlations. Selection of correlations at admission reported here, at discharge correlations were higher	Adequate (SF-36 some properties SF-36 reported but not for study population)
Tuntland [6]	Correlation of baseline scores between COPM and comparators (various single-item or sumscores); using Spearman’s rho	SPPB	EQ-5D, SOC-13, MHC-SF	Low with SPPB function, SPPB gait, SOC-13, MHC-SF, EQ-5D vas; moderate with EQ-5D activities	Low with SOC-13, MHC-SF	Eight predefined hypotheses, with expected correlations low or low/moderate; and validity assumed adequate when > 75% of hypotheses confirmed	Yes, 100% of hypotheses confirmed		Very good

*ACS-NL* Activity Card Sort-version Netherlands (NL) with ACS-Performance and ACS-Satisfaction, *AIMS2* Arthritis Impact Measurement Scales 2 (health-related QoL), *AUSCAN* Australian/Canadian Osteoarthritis Hand Index (pain, stiffness, physical disability), *BASDAI* Bath Ankylosing Spondylitis Disease Activity Index, *BASFI* Bath Ankylosing Spondylitis Functional Index, *BASMI* Bath Ankylosing Spondylitis Metrology Index, *BI* Barthel Index, *EQ-5D* Euroqol-5 dimensions, *EQ-5D-5L* Euroqol-5 domain-5 level questionnaire with EQ-5D-vas (health), *FAI* Frenchay Activities Index, *FIM* Functional Independence Measure, *MHAQ* Modified Stanford Health Assessment Questionnaire (disability ADL), *MHC-SF* Mental Health Continuum-Short Form (positive mental health), *N/A* not applicable, *OSA* Occupational Self-Assessment with OSA-Competences OSA-Values OSA-Priorities for change Rankin Scale [‘a global functional health index with a strong accent on physical disability’], *SA-SIP30* Stroke Adapted Sickness Impact Profile-30, *SF-36* Short form 36-item, *OPIs* occupational performance issues, *PD* Parkinson’s disease, *SF-36-P* Performance scale of SF-36, *SOC-13* Sense of Coherence questionnaire (coping), *SPPB* Short Physical Performance Battery, *vas* visual analog scale; *vs* versus, *WHO-5* 5-Item WHO well-being index, *WOMAC* Western Ontario and McMaster Universities Osteoarthritis Index (pain, stiffness, function)

Methodological quality was rated as very good or adequate for five studies. However, one of these studies did not use any preformulated hypotheses [18]. In the other four studies, the hypotheses of the authors were confirmed for COPM-P and COPM-S [6, 14, 15, 19]. Tuntland et al. concluded that construct validity was adequate as results were 100% in line with their eight hypotheses based on four other instruments. Poerbodipoero et al. confirmed discriminative validity, as COPM-scores differed between patients with mild and moderate Parkinson’s disease [15]. Edwards et al. and Roe et al. had no hypotheses regarding the size of expected correlations, but only examined whether correlations were ‘statistically significant’ to indicate adequate construct validity [14, 19].

In the remaining studies, methodological quality was rated inadequate or doubtful, because measurement properties of the comparator instruments were not reported (see supplement 3 table S3.2). Still, results of Enemark Larsen et al. are particularly interesting, as they conducted comparisons with OSA, which, like the COPM, scores values, and priorities: however, hypotheses of moderate correlations with OSA were not confirmed [13].

### Responsiveness

Responsiveness was reported in seven studies; see Table 3. The first COPM was assessed during intake or (the week of) admission, or 3–5 days after surgery [19]. Follow-up varied roughly from 3 weeks to 4 months.

**Table 3:** Summary of findings on responsiveness (*n* = 7 studies)

Author	Responsiveness, method used	Intervention and follow-up time^a^	Results	Results change scores COPM-P	Results change scores COPM-S	Hypothesis of authors	Hypotheses confirmed?	Remarks	Methodologic quality (item with lowest score)
Edwards et al. [19]	Construct approach comparator; comparison of delta COPM-P with delta WOMAC physical function (Pearson’s correlation and SRM, with bootstrapping)	Time between 3 and 5 days after surgery (T1) to 3 months after discharge (T4); *change expected*	Change for COPM-P was 4.6 (2.2–6.8), and for COPM-S 4.3 (2.7–7.0). Correlation of change scores between COPM-P and WOMAC physical function −0.54; SRM for COPM-P 1.77 and for WOMAC pf 1.89, with difference 0.12 (n.s.)	Change scores correlated high with WOMAC physical function		Hypothesis that change scores of measures intended for a similar purpose should display moderate correlation. No hypothesis for SRM on expected magnitude of effect size	Yes, moderate (borderline high) correlations	In publication responsiveness is called *‘longitudinal convergent construct validity’*	Adequate (properties WOMAC for present population not reported in text)
Enemark Larsen [20]	Construct approach comparator and subgroups, comparison of delta COPM with delta WHO-5, EQ VAS (using Spearman correlations), and in subgroups of AQ with self-rated change, and in subgroups with cut-points WHO-5 and EQ VAS (using ROC curves)	Rehabilitation for various reasons; follow-up assessment after mean 101 days (SD 59.1; range 7–288)	Correlations of change scores of COPM-P and -S with WHO-5 0.22 and 0.32, with EQ VAS 0.32 and 0.36; Mean change scores COPM-P 3.1 (SD 2.8), COPM-S 3.0 (SD 1.8); changes higher in better subgroups for AQ/sharp (AUC 0.86/0.76 for P and 0.85/0.75 for S), for WHO-5 only the higher end, for EQ VAS only the lower end with COPM-P (AUCs < 0.70)	Change scores correlated low with WHO-5 and EQ VAS; mean changes higher in better subgroups AQ (AUC > 0.70)	Change scores correlated low with WHO-5 and EQ VAS; mean changes higher in better subgroups AQ (AUC > 0.70)	Hypotheses formulated based on anchor question self-rated change, and based on clinically relevant cut-points for comparator scales	Partly, COPM responsive to change compared to patient-reported improvement, but not so much with WHO-5 or EQ VAS	COPM-sumscores not limited to similar OPIs across both time points; MIC recommendations	C: adequate/s: doubtful (no description of important characteristics subgroups)
Kjeken [22]	Construct approach intervention with comparators; analyses of change scores for COPM using paired sample *t* test, and comparison of standardized response means (SRM) with other instruments MHAQ WOMAC AUSCAN	OT intervention (depending on intake), follow-up 4 months after baseline	Mean change scores COPM-P −1.51 (CI −2.04; −0.98), COPM-S −2.22 (CI −2.80; −1.63); SRM large for COPM-P (0.7) and COPM-S (0.9), moderate for MHAQ (0.5), and small for WOMAC (0.15) and AUSCAN (0.25)	Scores changed significantly; SRM higher than for other measures MHAQ, WOMAC, AUSCAN	Scores changed significantly; SRM higher than for other measures MHAQ, WOMAC, AUSCAN	No hypotheses regarding treatment effect	N/A		Doubtful (patients who changed medication or undergone surgical treatment were excluded (14 of 79))
Roe [14]	Construct approach intervention, difference in COPM-scores between admission and discharge (one sample *t* test, with bootstrapping)	Admission to sub-acute care unit, with average length of stay 17 days	Change scores not presented, only p values (*p* = 0.001), and means at admission and discharge. We deducted that at discharge COPM-P scores were lower (3.846−3.710 = delta−0.136) and COPM-S higher (7.374−7.518 = delta 0.144)	Scores changed significantly but not meaningful (< 0.5 pt) and in wrong direction	Scores changed significantly but not meaningful (< 0.5 pt)	It was hypothesized that there would be a statistically significant difference between admission and discharge for both COPM-P and COPM-S scores. No hypotheses on size of difference	Responsiveness of COPM-P and COPM-S scales cannot be confirmed	Deducted change scores of COPM-P and COPM-S are not in the same direction	Inadequate (statistical method applied not appropriate, with *t* test only measure of significant change not valid change)
Thyer [18]	Construct approach comparator, comparison delta COPM with delta FIM and SF-36 (Pearson’s correlation, with bootstrapping)	OT treatment, mean length of stay 21.8 days (SD 13.5); follow-up within 48 h before discharge; *change expected*	Correlation of change scores between COPM-P and FIM total 0.637 phys 0.601 cogn 0.428, SF-36 phys 0.225 ns mental 0.124; between COPM-S and FIM total 0.550 phys 0.518 cogn 0.427, SF-36 phys 0.164 ns mental 0.130	Change scores correlated high with FIM tot and phys, moderate with FIM cogn, and low with SF-36 phys and mental	Change scores correlated high with FIM phys, but low with SF-36 phys	No hypotheses	N/A	In publication responsiveness is called *‘construct validity of change scores’*	Adequate (SF-36 unclear if properties apply to study pop; unclear if bootstrapping method is allowed (*n* = 36))
Tuntland [6]	Construct approach comparator and subgroups; comparison of delta COPM with delta SPPB, EQ-5D self-care, SOC-13, MHC-SF (using Pearson’s correlation), and in subgroups of GRS on self-reported change (using independent sample *t* test)	Intervention group of trial, receiving 10 weeks reablement; *change expected*	Correlation of change scores between COPM-P and SPPB 0.40; mean difference in COPM-P-scores for GRS (no vs little) −1.45, for GRS (little vs much) −1.53; and for COPM-S −1.12 n.s. and −1.61; 63% (5/8) of hypotheses confirmed	Change scores correlated moderate with SPPB; scores changed significantly (~ 1.5 pt) in GRS-subgroups	Scores changed significantly (~ 1.5 pt) in GRS-subgroups [little vs much], but n.s. and less (~ 1 pt) in GRS-subgroups [no vs little]	Hypotheses for Global rating scale (4×), SPPB, EQ-5D, SOC-13 and MHC-SF; significant differences expected between GRS-subgroups; low correlations Expected with change scores of comparator instruments	Yes, ‘moderate’ given 63% of hypotheses were confirmed	MIC recommendations	Adequate (assuming that independent sample *t* test is comparable to ANOVA)
Wressle [21]	Construct approach intervention, median change scores between begin and end of rehabilitation period (Wilcoxon’s signed-rank test for ordinal scores)	Geriatric, orthopedic or neurologic rehabilitation; with follow-up assessment after median 23 days (range 2–262); *change expected*	At the end of the intervention, median change scores were for COPM-P 3 (range 2–6) and for COPM-S 4 (range 2–6); for subgroup inpatients (*n* = 70) medians were 3 and 4	Meaningful change in score (> 2 pt) after intervention, responsive instrument	Meaningful change in score (> 2 pt) after intervention, responsive instrument	No hypotheses formulated on expected difference	N/A	Responsiveness per problem type not reported here, as statistics were doubtful	Doubtful (poor description of intervention given, level of testing)

*AUSCAN* Australian/Canadian Osteoarthritis Hand Index (pain, stiffness, physical disability), *AQ* anchor-based question, *EQ VAS* EuroQol Visual Analogue Scale, *FIM* Functional Independence Measure, *GRS* Global Rating Scale, *MHAQ* modified Stanford Health Assessment Questionnaire (disability ADL), *SF-36* short form 36-item, *SOC-13* Sense of Coherence questionnaire (coping), *SPPB* Short Physical Performance Battery, *WOMAC* Western Ontario and McMaster Universities Osteoarthritis Index (pain, stiffness, physical disability)

^a^Information to decide whether we know (or assume) on forehand an effect of the intervention and thus effect size

Four studies used a ‘construct approach comparator’: they compared delta scores on COPM with delta scores on another instrument, using correlations [18–20] or predefined hypotheses [6]. Methodological quality scored adequate in these four studies. In three studies, predefined hypotheses were (partly) confirmed, showing moderate responsiveness. For change scores on COPM-P, high correlations were found with change in WOMAC physical function and FIM total and physical scores. Moderate correlations were observed with changes in FIM cognitive scores and SPPB, and low correlations were observed with change in SF-36 physical and mental scores and WHO-5 and EQ VAS. Tuntland et al. and Enemark Larsen et al. also compared COPM change scores in subgroups with no/little/much improvement based on an anchor question on patients’ impression of change, and both found relevant differences between groups, especially in COPM-P scores (e.g., for Tuntland et al. 1.5 pt between subgroups little versus much change). As Enemark Larsen et al. did not describe characteristics of the subgroups, methodological quality was rated doubtful.

Three studies used a ‘construct approach intervention’ and analyzed post-intervention change scores [14, 21], and one study also compared standardized response measures (SRM) with other instruments [22]. Methodological quality for these studies was doubtful/inadequate. Only Roe et al. formulated hypotheses beforehand; however, these were based on statistical significance and not on the size of the effect. The COSMIN manual advises against interpretation of results when no hypotheses about the size of the effect are formulated in advance. However, a meaningful change (that is > 2 pt, as defined by the authors of the COPM) in COPM-scores was observed in two studies, suggesting good responsiveness.

### Reliability

Reliability was reported on in three publications with four studies [16, 23, 24]; see Table 4. In the study of Cup et al., assessments took place 2–6 months post-stroke, in Enemark Larsen et al. 5 years after surgery, and in Sewell et al. at the start of the pulmonary rehabilitation program. There was a high correlation between test–retest scores, measured on average after 1 week, showing a good reliability of the COPM. Methodological quality was adequate in the study of Sewell et al. However, in the study of Cup et al., methodological quality was considered doubtful, as no intraclass correlation coefficient (ICC) was calculated, and it was unclear whether patients remained stable between tests. Of interest, Cup et al. reported that 56% of problems identified during the first COPM were also present during the second assessment.

**Table 4:** Summary of findings on reliability (*n* = 4 studies)

Author	Reliability, method	Results	Results COPM-P	Results COPM-S	Conclusion	Remarks	Methodological quality (item with lowest score)
Cup et al. [16]	Interviews 6 (*n* = 24) or 2 (*n* = 2) months post-stroke; Comparison of test–retest scores within mean 8 days (range 5–16 days); Spearman’s Rho correlation coefficients	Correlations of scores for COPM-P 0.89 and for COPM-S 0.88 (both *p* < 0.001)	High correlation between test–retest scores	High correlation between test–retest scores	Good test–retest reliability for scores (but moderate stability of COPM item pool)	In 56% identification of same problems, thus the ‘item pool’ is not completely stable	Doubtful (unclear whether patients were stable; no ICC but Spearman correlation)
Enemark Larsen [13] Study A (2R)	Interviewed twice with mean interval of 10 days (SD 5.9; range 2–27). Comparison of scores between raters; ICC based on random effect model (without *p* values); limits of agreement (LoA) and coefficient of repeatability (CR)	ICCs based on all OPIs were for COPM-I 65.2 (CI 52.0–76.4), for COPM-P 58.9 (CI 44.4–71.9), for COPM-S 51.4 (35.9–66.6). Bland–Altman plots show no systematic change	Moderate ICC	Moderate ICC	Moderate inter-rater reliability	Overlapping items (OPIs) 39.1%	Adequate (information in appendix)
Enemark Larsen [13] Study B (1R)	Interviewed twice with mean interval of 10 days (SD 3.3; range 4–18). Comparison of test–retest scores; ICC based on random effect model (without *p* values); limits of agreement (LoA) and coefficient of repeatability (CR)	ICCs based on all OPIs were for COPM-I 49.4 (CI 32.2–66.8), for COPM-P 75.3 (CI 63.6–84.2), for COPM-S 70.3 (CI 57.0–80.8). Bland–Altman plots show no systematic change	Good ICC	Good ICC	Good test–retest reliability	Overlapping items (OPIs) in 43.9%; we used ICC cut-off from COSMIN (> 0.70) instead of publication (> 0.75)	Adequate (information in appendix)
Sewell [24]	Interviews at start of pulmonary rehabilitation program. Comparison of test–retest scores, with reassessment after approx. 7 days by the same occupational therapist; analyses of mean difference and ICC between tests; spread of differences with Bland–Altman plots	Mean difference with 95% CI: for COPM-P 0.14 (− 0.39 to 0.68), for COPM-S 0.42 (− 0.18 to 1.01); with ICC *r* = 0.92 and *r* = 0.90. Bland–Altman plots show no systematic change, although for COPM-S clinical relevant difference (> 2) between ratings in 2 of 15 patients	High ICC; no systematic change	High ICC; no systematic change	Good test–retest reliability, COPM is a reproducible measure in clients with COPD	Scores possibly based on different problems for test 1 and test 2 (no selection of ‘same item pool’)	Adequate

## Discussion

In this systematic literature review, COPM showed moderate inter-rater reliability, good test–retest reliability, and good content validity in GR patients. For construct validity, 4 studies with minimal risk of bias showed good construct validity. In 5 other studies, either considerable risk of bias was present, or the authors did not formulate hypotheses for their comparisons between instruments, which hampered our ability to interpret the results. Responsiveness was found to be moderate in the 3 studies scoring adequate for methodological quality.

We found three earlier literature reviews on psychometric properties of the COPM. Yang et al. included three psychometric studies on patients with stroke [25]. Nieuwenhuizen et al. included an extensive overview of the literature (25 studies up to 2014) in the introduction of their publication, without reporting on their search methods or age of study populations [26]. Carswell et al. systematically included 19 studies up to 2004 with various populations and concluded that the COPM is a valid, reliable, clinically useful and responsive outcome measure [7]. The latter review included findings from different kind of settings, and studies were not critically appraised on methodological quality. In the present systematic review, we focused specifically on the GR setting and took a more rigorous approach. We included 9 different studies compared to the previous reviews (8 studies after 2014), thus providing more up-to-date and substantiated results. Two researchers extracted results and scored risk of bias independently following COSMIN guidelines [9]. This is a very strict method, as the item with the lowest score defines the methodological quality.

For each property of the COPM, we found at least one study in the geriatric rehabilitation setting. In general, this gave sufficient evidence, although the number or quality of studies that is needed as evidence has been a point of discussion [27]. We chose to include studies of patients with an average age of 60 years and older, which some might consider too low for geriatric rehabilitation as in a EU survey most referred patients were older than 70 years [28]. However, we followed the European consensus statement that recommends focusing on frailty rather than age when referring patients to geriatric rehabilitation [1, 2]. If we had limited ourselves to a mean/median age of 70 years and older, our conclusions would have been the same (based on 6 studies) except for inter-rater reliability (no study ≥ 70 years). Two of the ten included studies were in a home-based setting. Although this was geriatric rehabilitation, this population is probably cognitively less impaired than patients in institutional settings.

It is noteworthy that a large variety of instruments was used in the comparison with COPM-scores to determine construct validity. Not only measures of physical functioning, but also instruments for quality of life, mental functioning, impact of sickness, and coping were used. Moreover, we found that for the same (type of) instruments, hypotheses among authors varied considerably (see Table 5). For instance, while Roe et al. expected COPM-scores to be related to SF-36 physical functioning scores, Cup et al. hypothesized that COPM-scores would be unrelated to the Barthel Index. This underlines the lack of consensus among researchers and clinicians regarding what COPM-scores can tell us, and it shows that its construct is ambiguous. This is also the reason why we chose not to formulate our own construct hypotheses and interpret study results accordingly for this review, as COSMIN suggests. Also, it is questionable whether studying divergent construct validity is informative. Three studies finding low correlations between the COPM and other measures concluded that these confirmed their hypotheses regarding construct validity [6, 16, 22], because the COPM is an individual (patient-unique) measure, and low correlation would support the notion that COPM provides information that is not obtainable with other standardized measures. Cup et al. added that only 25% of problems reported in the COPM were present in the standardized measures. However, these low correlations merely confirm what the COPM does not measure and do not tell us anything about the construct that the COPM does measure.

**Table 5:** Overview of comparator instruments used in studies for construct validity, with predefined hypotheses of authors. Instruments are in alphabetical order, for physical functioning and other constructs

Comparator instruments used for construct validity	COPM-performance: hypothesis of authors	COPM-satisfaction: hypothesis of authors	Studies
**Physical functioning**			
ACS-performance	Moderate		Poerbodipoero [15]
ACS-satisfaction		Moderate	Poerbodipoero [15]
AUSCAN function	Low/moderate	Low/moderate	Kjeken [22]
BI	‘Unrelated’		Cup et al. [16]
EQ-5D item mobility	Low		Enemark Larsen [13]
EQ-5D item usual activities	Low/moderate; low		Tuntland[6]; Enemark Larsen [13]
EQ-5D item self-care	Low		Enemark Larsen [13]
FAI	‘Unrelated’		Cup et al. [16]
FIM motor	‘Related’; none	‘Related’; none	Roe [14]; Thyer [18]
FIM total	‘Related’; none	‘Related’; none	Roe[14]; Thyer [18]
MHAQ	Low/moderate	Low/moderate	Kjeken [22]
OSA competence	Moderate		Enemark Larsen [13]
OSA values	Moderate		Enemark Larsen [13]
SF-36 physical	‘Related’; none	‘Related’; none	Roe [14]; Thyer [18]
SPPB item gait test	Low/moderate		Tuntland [6]
SPPB sum score	Low		Tuntland [6]
WOMAC function	‘Related’; low/moderate	‘Related’; low/moderate	Edwards et al. [19], Kjeken [22]
**Other constructs**			
Disease activity (vas)	‘Related’	‘Related’	Kjeken [22]
EQ-5D item anxiety		Low	Enemark Larsen [13]
EQ-5D item pain		Low	Enemark Larsen [13]
EQ-5D total score	‘Unrelated’		Cup et al. [16]
EQ-5D vas	Low/moderate; low		Tuntland[6]; Enemark Larsen [13]
FIM cognitive	‘Related’; none	‘Related’; none	Roe[14]; Thyer [18]
MHC-SF	Low	Low	Tuntland [6]
Rankin Scale	‘Unrelated’		Cup et al. [16]
SA-SIP30	‘Unrelated’		Cup et al. [16]
SF-36 mental	‘Related’; none	‘Related’; none	Roe[14]; Thyer [18]
SOC-13	Low	Low	Tuntland [6]
WHO-5	Low		Enemark Larsen [13]

*ACS-NL* Activity Card Sort-version Netherlands (NL), with ACS-Performance and ACS-Satisfaction, *AUSCAN* Australian/Canadian Osteoarthritis Hand Index (pain, stiffness, physical disability), *BI* Barthel Index, *EQ-5D* Euroqol-5 dimensions, *EQ-5D-5L* Euroqol-5 dimension-5 level questionnaire, EQ-5D-vas (health), *FAI* Frenchay Activities Index; *FIM* Functional Independence Measure, *MHAQ* modified Stanford Health Assessment Questionnaire (disability ADL), *MHC-SF* Mental Health Continuum-Short Form (positive mental health), OSA Occupational Self-Assessment, with OSA-Competences OSA-Values OSA-Priorities for change; Rankin Scale [‘a global functional health index with a strong accent on physical disability’], *SA-SIP30* Stroke Adapted Sickness Impact Profile-30, *SF-36* Short Form Survey 36-item, *SOC-13* Sense of Coherence questionnaire (coping), *SPPB* Short Physical Performance Battery, *WHO-5 *5-item WHO well-being index, *WOMAC* Western Ontario and McMaster Universities Osteoarthritis Index (pain, stiffness, function)

Looking beyond measurement properties, various studies examining feasibility and clinical utility of the COPM found that implementation of the COPM in practice is not without difficulty. Challenges regarding scoring are often mentioned, in particular for patients with cognitive impairment [6, 14, 22, 29]. Especially, older clients may not be familiar with the use of scales, or understand their meaning. As the COPM leaves room to be performed in personalized styles, this ability to score the instrument may depend in part on the interviewing skills of the occupational therapist as well. Kjeken et al. also reported problems with clients’ ability to perceive the difference between satisfaction and performance scores [22]. This is in line with our observation that studies report COPM-P and COPM-S scores in the same range. In a literature review [30] and in qualitative research [14, 29] it was concluded that the COPM ensures a client-centered approach and facilitates client engagement. Especially in geriatric rehabilitation, this is important, because more than in rehabilitation for younger persons, GR is about finding a new balance, often with a higher degree of dependency, while trying to preserve autonomy and self-management. Kjeken et al. found that some patients are anxious about being an active participant in the treatment process, but remarked that this in itself can be valuable information for therapists [22]. In conclusion, training and a good introduction to the COPM seem necessary, as the therapist must develop a client-centered approach.

Structured assessments by healthcare professionals are important to evaluate rehabilitation progress. Our results show that COPM-scores may play a role in the evaluation of geriatric rehabilitation on an individual level. We found that responsiveness was moderate in three studies that scored adequate for methodological quality. Worth mentioning is that Tuntland et al. and Enemark Larsen et al. studied interpretability, and both recommended a higher cut-off point for minimal important change, using, respectively, 3 or 3.5 instead of 2 points mentioned in the COPM-manual [6, 20].

However, it is still uncertain whether aggregated (average) COPM-scores from various departments or care organizations can be used in benchmarking. Unfortunately, for this purpose, evaluation of geriatric rehabilitation is mostly based on the easily available parameters such as length of stay and costs.

To conclude, the use of the COPM will give occupational therapists and the multidisciplinary team information that is relevant for geriatric rehabilitation, as shown by the study on content validity. This can help to make treatment more personalized and client-centered. Also, the progress of the rehabilitation can be evaluated, because the COPM-scores can be assessed reliably and are responsive to change. And although there were many studies on construct validity, authors had different opinions on exactly what COPM-scores tell us, as they used a variety of comparator instruments and different hypotheses. As such, consensus on exact interpretation of the scores is needed, especially of aggregated (average) scores outside the context of direct patient care, e.g., when comparing groups of patients in research or in benchmarking.

## Supplementary Information

Below is the link to the electronic supplementary material.

Supplementary file1 (DOC 148 KB)

Supplementary file2 (DOCX 49 KB)

Supplementary file3 (DOCX 23 KB)
