# Supplementary material for: Systematic review of measurement properties of the Canadian Occupational Performance Measure in geriatric rehabilitation
Source: Eur Geriatr Med. 2022 Aug 23;13(6):1281–98. doi: 10.1007/s41999-022-00692-8 (PMC9722840; doi:10.1007/s41999-022-00692-8)
Supplement: Supplementary file 1 — Supplementary file1 (DOC 148 KB) [file 41999_2022_692_MOESM1_ESM.doc]

Search of publications for systematic review ’Properties of the COPM in geriatric rehabilitation’:

Search for publications on COPM (canadian occupational performance scale) AND older people excluding Meeting abstract references.

The search was performed by a librarian of the LUMC on **28th of March 2019**, and updated on the 22th of September 2020, and again updated on the 15^th^ of March 2022.

Databases:

**PubMed**

<http://www.ncbi.nlm.nih.gov/pubmed?otool=leiden>

<https://pmlegacy.ncbi.nlm.nih.gov/>

(("canadian occupational performance"[tw]) **AND ("Aged"[mesh] OR "elderly"[tw] OR "elder"[tw] OR "elders"[tw] OR geriatr*[tw] OR "Homes for the Aged"[mesh] OR "Health Services for the Aged"[mesh] OR "Senior Centers"[mesh] OR older person*[tw] OR old person*[tw] OR older patient*[tw] OR old patient*[tw] OR "older women"[tw] OR "old women"[tw] OR "older men"[tw] OR "old men"[tw] OR old adult*[tw] OR older adult*[tw] OR "Older individual"[tw] OR "Older individuals"[tw] OR "old people"[tw] OR "older people"[tw] OR "Oldest Old"[tw] OR "Nonagenarians"[tw] OR "Nonagenarian"[tw] OR "Octogenarians"[tw] OR "Octogenarian"[tw] OR "Centenarians"[tw] OR "Centenarian"[tw] OR "septuagenarian"[tw] OR "septuagenarians"[tw] OR "Aging"[mesh] OR "aging"[tw] OR "ageing"[tw] OR "older population"[tw] OR "aging population"[tw] OR "aging population"[tw] OR geront*[tw] OR "old-aged"[tw] OR "old-age"[tw] OR "old aged"[tw] OR "old age"[tw])) OR** (("canadian occupational performance"[ti]) **AND ("Adult"[mesh]))**

**Embase**

<http://ovidsp.ovid.com/ovidweb.cgi?T=JS&PAGE=main&MODE=ovid&D=oemezd>

(("canadian occupational performance".mp) **AND (exp "Aged"/ OR "elderly".mp OR "elder".mp OR "elders".mp OR geriatr*.mp OR exp "Elderly Care"/ OR exp "Senior Center"/ OR older person*.mp OR old person*.mp OR older patient*.mp OR old patient*.mp OR "older women".mp OR "old women".mp OR "older men".mp OR "old men".mp OR old adult*.mp OR older adult*.mp OR "Older individual".mp OR "Older individuals".mp OR "old people".mp OR "older people".mp OR "Oldest Old".mp OR "Nonagenarians".mp OR "Nonagenarian".mp OR "Octogenarians".mp OR "Octogenarian".mp OR "Centenarians".mp OR "Centenarian".mp OR "septuagenarian".mp OR "septuagenarians".mp OR exp "Aging"/ OR "aging".mp OR "ageing".mp OR "older population".mp OR "aging population".mp OR "aging population".mp OR geront*.mp OR "old-aged".mp OR "old-age".mp OR "old aged".mp OR "old age".mp)) NOT (conference review or conference abstract).pt**

**Web of Science**

<http://isiknowledge.com/wos>

ts=(("canadian occupational performance") **AND ("elderly" OR "elder" OR "elders" OR geriatr* OR "Elderly Care" OR "Senior Center" OR "older person*" OR "old person*" OR "older patient*" OR "old patient*" OR "older women" OR "old women" OR "older men" OR "old men" OR "old adult*" OR "older adult*" OR "Older individual" OR "Older individuals" OR "old people" OR "older people" OR "Oldest Old" OR "Nonagenarians" OR "Nonagenarian" OR "Octogenarians" OR "Octogenarian" OR "Centenarians" OR "Centenarian" OR "septuagenarian" OR "septuagenarians" OR "Aging" OR "aging" OR "ageing" OR "older population" OR "aging population" OR "aging population" OR geront* OR "old-aged" OR "old-age" OR "old aged" OR "old age")) NOT dt=(meeting abstract)**

**Cochrane**

<https://www.cochranelibrary.com/advanced-search/search-manager>

(("canadian occupational performance") **AND ("elderly" OR "elder" OR "elders" OR geriatr* OR "Elderly Care" OR "Senior Center" OR "older person*" OR "old person*" OR "older patient*" OR "old patient*" OR "older women" OR "old women" OR "older men" OR "old men" OR "old adult*" OR "older adult*" OR "Older individual" OR "Older individuals" OR "old people" OR "older people" OR "Oldest Old" OR "Nonagenarians" OR "Nonagenarian" OR "Octogenarians" OR "Octogenarian" OR "Centenarians" OR "Centenarian" OR "septuagenarian" OR "septuagenarians" OR "Aging" OR "aging" OR "ageing" OR "older population" OR "aging population" OR "aging population" OR geront* OR "old-aged" OR "old-age" OR "old aged" OR "old age")):ti,ab,kw NOT (conference abstract):pt**

**Emcare** <http://ovidsp.ovid.com/ovidweb.cgi?T=JS&NEWS=n&CSC=Y&PAGE=main&D=emcr>

(("canadian occupational performance".mp) **AND (exp "Aged"/ OR "elderly".mp OR "elder".mp OR "elders".mp OR geriatr*.mp OR exp "Elderly Care"/ OR exp "Senior Center"/ OR older person*.mp OR old person*.mp OR older patient*.mp OR old patient*.mp OR "older women".mp OR "old women".mp OR "older men".mp OR "old men".mp OR old adult*.mp OR older adult*.mp OR "Older individual".mp OR "Older individuals".mp OR "old people".mp OR "older people".mp OR "Oldest Old".mp OR "Nonagenarians".mp OR "Nonagenarian".mp OR "Octogenarians".mp OR "Octogenarian".mp OR "Centenarians".mp OR "Centenarian".mp OR "septuagenarian".mp OR "septuagenarians".mp OR exp "Aging"/ OR "aging".mp OR "ageing".mp OR "older population".mp OR "aging population".mp OR "aging population".mp OR geront*.mp OR "old-aged".mp OR "old-age".mp OR "old aged".mp OR "old age".mp))**

**PsycINFO**

<http://search.ebscohost.com/login.aspx?authtype=ip,uid&profile=lumc&defaultdb=psyh>

TI(("canadian occupational performance") **AND ("elderly" OR "elder" OR "elders" OR geriatr* OR "Elderly Care" OR "Senior Center" OR "older person*" OR "old person*" OR "older patient*" OR "old patient*" OR "older women" OR "old women" OR "older men" OR "old men" OR "old adult*" OR "older adult*" OR "Older individual" OR "Older individuals" OR "old people" OR "older people" OR "Oldest Old" OR "Nonagenarians" OR "Nonagenarian" OR "Octogenarians" OR "Octogenarian" OR "Centenarians" OR "Centenarian" OR "septuagenarian" OR "septuagenarians" OR "Aging" OR "aging" OR "ageing" OR "older population" OR "aging population" OR "aging population" OR geront* OR "old-aged" OR "old-age" OR "old aged" OR "old age")) OR (**TI("canadian occupational performance") **AND SU("elderly" OR "elder" OR "elders" OR geriatr* OR "Elderly Care" OR "Senior Center" OR "older person*" OR "old person*" OR "older patient*" OR "old patient*" OR "older women" OR "old women" OR "older men" OR "old men" OR "old adult*" OR "older adult*" OR "Older individual" OR "Older individuals" OR "old people" OR "older people" OR "Oldest Old" OR "Nonagenarians" OR "Nonagenarian" OR "Octogenarians" OR "Octogenarian" OR "Centenarians" OR "Centenarian" OR "septuagenarian" OR "septuagenarians" OR "Aging" OR "aging" OR "ageing" OR "older population" OR "aging population" OR "aging population" OR geront* OR "old-aged" OR "old-age" OR "old aged" OR "old age")) OR (**TI("canadian occupational performance") **AND KW("elderly" OR "elder" OR "elders" OR geriatr* OR "Elderly Care" OR "Senior Center" OR "older person*" OR "old person*" OR "older patient*" OR "old patient*" OR "older women" OR "old women" OR "older men" OR "old men" OR "old adult*" OR "older adult*" OR "Older individual" OR "Older individuals" OR "old people" OR "older people" OR "Oldest Old" OR "Nonagenarians" OR "Nonagenarian" OR "Octogenarians" OR "Octogenarian" OR "Centenarians" OR "Centenarian" OR "septuagenarian" OR "septuagenarians" OR "Aging" OR "aging" OR "ageing" OR "older population" OR "aging population" OR "aging population" OR geront* OR "old-aged" OR "old-age" OR "old aged" OR "old age")) OR (**TI("canadian occupational performance") **AND AB("elderly" OR "elder" OR "elders" OR geriatr* OR "Elderly Care" OR "Senior Center" OR "older person*" OR "old person*" OR "older patient*" OR "old patient*" OR "older women" OR "old women" OR "older men" OR "old men" OR "old adult*" OR "older adult*" OR "Older individual" OR "Older individuals" OR "old people" OR "older people" OR "Oldest Old" OR "Nonagenarians" OR "Nonagenarian" OR "Octogenarians" OR "Octogenarian" OR "Centenarians" OR "Centenarian" OR "septuagenarian" OR "septuagenarians" OR "Aging" OR "aging" OR "ageing" OR "older population" OR "aging population" OR "aging population" OR geront* OR "old-aged" OR "old-age" OR "old aged" OR "old age"))**

**Academic Search Premier [fulltextzoeken]**

<http://search.ebscohost.com/login.aspx?authtype=ip,uid&profile=lumc&defaultdb=aph>

TI(("canadian occupational performance") **AND ("elderly" OR "elder" OR "elders" OR geriatr* OR "Elderly Care" OR "Senior Center" OR "older person*" OR "old person*" OR "older patient*" OR "old patient*" OR "older women" OR "old women" OR "older men" OR "old men" OR "old adult*" OR "older adult*" OR "Older individual" OR "Older individuals" OR "old people" OR "older people" OR "Oldest Old" OR "Nonagenarians" OR "Nonagenarian" OR "Octogenarians" OR "Octogenarian" OR "Centenarians" OR "Centenarian" OR "septuagenarian" OR "septuagenarians" OR "Aging" OR "aging" OR "ageing" OR "older population" OR "aging population" OR "aging population" OR geront* OR "old-aged" OR "old-age" OR "old aged" OR "old age")) OR (**TI("canadian occupational performance") **AND SU("elderly" OR "elder" OR "elders" OR geriatr* OR "Elderly Care" OR "Senior Center" OR "older person*" OR "old person*" OR "older patient*" OR "old patient*" OR "older women" OR "old women" OR "older men" OR "old men" OR "old adult*" OR "older adult*" OR "Older individual" OR "Older individuals" OR "old people" OR "older people" OR "Oldest Old" OR "Nonagenarians" OR "Nonagenarian" OR "Octogenarians" OR "Octogenarian" OR "Centenarians" OR "Centenarian" OR "septuagenarian" OR "septuagenarians" OR "Aging" OR "aging" OR "ageing" OR "older population" OR "aging population" OR "aging population" OR geront* OR "old-aged" OR "old-age" OR "old aged" OR "old age")) OR (**TI("canadian occupational performance") **AND KW("elderly" OR "elder" OR "elders" OR geriatr* OR "Elderly Care" OR "Senior Center" OR "older person*" OR "old person*" OR "older patient*" OR "old patient*" OR "older women" OR "old women" OR "older men" OR "old men" OR "old adult*" OR "older adult*" OR "Older individual" OR "Older individuals" OR "old people" OR "older people" OR "Oldest Old" OR "Nonagenarians" OR "Nonagenarian" OR "Octogenarians" OR "Octogenarian" OR "Centenarians" OR "Centenarian" OR "septuagenarian" OR "septuagenarians" OR "Aging" OR "aging" OR "ageing" OR "older population" OR "aging population" OR "aging population" OR geront* OR "old-aged" OR "old-age" OR "old aged" OR "old age")) OR (**TI("canadian occupational performance") **AND AB("elderly" OR "elder" OR "elders" OR geriatr* OR "Elderly Care" OR "Senior Center" OR "older person*" OR "old person*" OR "older patient*" OR "old patient*" OR "older women" OR "old women" OR "older men" OR "old men" OR "old adult*" OR "older adult*" OR "Older individual" OR "Older individuals" OR "old people" OR "older people" OR "Oldest Old" OR "Nonagenarians" OR "Nonagenarian" OR "Octogenarians" OR "Octogenarian" OR "Centenarians" OR "Centenarian" OR "septuagenarian" OR "septuagenarians" OR "Aging" OR "aging" OR "ageing" OR "older population" OR "aging population" OR "aging population" OR geront* OR "old-aged" OR "old-age" OR "old aged" OR "old age"))**
