# Supplementary material for: Systematic review of measurement properties of the Canadian Occupational Performance Measure in geriatric rehabilitation
Source: Eur Geriatr Med. 2022 Aug 23;13(6):1281–98. doi: 10.1007/s41999-022-00692-8 (PMC9722840; doi:10.1007/s41999-022-00692-8)
Supplement: Supplementary file 2 — Supplementary file2 (DOCX 49 KB) [file 41999_2022_692_MOESM2_ESM.docx]

**Figure S2.1.** PRISMA Flow diagram of included studies.

**Identification of studies via databases and registers**

Records removed *before screening*:

Duplicate records removed (n=312)

Records marked as ineligible by

automation tools (n=0)

Records removed for other reasons

(n=0)

Records (n= 604) identified from:

Pubmed (n = 176)

Embase (n = 186)

Emcare (n = 111)

Web of Science (n = 72)

COCHRANE Library (n = 47)

Academic Search Premier(n=9)

PsychINFO (n = 3)

**Identification**

Records excluded, no reports on measurement properties (n = 249)

Records screened

(n = 292)

Reports not retrieved

(n = 0)

Reports sought for retrieval

(n = 43 publications)

**Screening**

Reports excluded:

Population mean or median age <60

(n=20)

No geriatric rehabilitation (n=2)

Reported properties (e.g. utility) not

of interest for this review (n=9)

Reports assessed for eligibility

(n = 43 publications)

Studies included in review

(n = 13 from 12 publications)

Reports of included studies

(n = 13 from 12 publications)

**Included**
