# Supplementary material for: Systematic review of measurement properties of the Canadian Occupational Performance Measure in geriatric rehabilitation
Source: Eur Geriatr Med. 2022 Aug 23;13(6):1281–98. doi: 10.1007/s41999-022-00692-8 (PMC9722840; doi:10.1007/s41999-022-00692-8)
Supplement: Supplementary file 3 — Supplementary file3 (DOCX 23 KB) [file 41999_2022_692_MOESM3_ESM.docx]

**Table S3.1.** Methodological quality for construct validity and responsiveness of included studies where applicable.

| **Author** | **Methodological quality**  **items for construct validity** | | | **Methodological quality**  **items for responsiveness** † | | | | | |
| --- | --- | --- | --- | --- | --- | --- | --- | --- | --- |
|  | Design: construct of comparator clear | Design: report of properties comparator | Statistical methods | Design (c s): construct of comparator clear | Design (c s): report of properties comparator | Design (s): description  characteristics  subgroups | Design (i): adequate description intervention | Statistical methods | Other |
| Cup 2003 | ☺ | ☹ | ☺ |  |  |  |  |  |  |
| Edwards 2007 | ☺ | 😐 | ☺ | ☺ | 😐 |  |  | ☺ | ☺ |
| Enemark Larsen 2020 | ☺ | ☹ | ☺ |  |  |  |  |  |  |
| Enemark Larsen 2022 |  |  |  | ☺ | 😐 | ? |  | ☺ | ☺ |
| Kjeken 2004 | ☺ | ☹ | ☺ |  |  |  | ☺ | ☺ | ? |
| Poerbodipoero 2016 a | ☺ | ? | ☺ |  |  |  |  |  |  |
| Poerbodipoero 2016 b | ☺ * | | ☺ |  |  |  |  |  |  |
| Roe 2020 | ☺ | 😐 | 😐 |  |  |  | ? | ☹ | ? |
| Thyer 2018 | ☺ | 😐 | 😐 | ☺ | 😐 |  |  | ☺ | 😐 |
| Tuntland 2016 | ☺ | ☺ | ☺ | ☺ | ☺ |  |  | 😐 | ☺ |
| Wressle 1999 |  |  |  |  |  |  | ? | ? | ☺ |

Legend: ☺ very good 😐 adequate ? doubtful ☹ inadequate

* Design ‘description of subgroup’ as analyses concerned comparison of subgroups.

† Responsiveness design info: construct approach with c = comparison, s = subgroup, i = intervention

| **Author** | **Content validity, method used** | **1.Relevance for construct, target population, and context of use** | **2.Comprehensiveness** | **3.Comprehensibility (including feasibility)** | **Methodological quality (item with lowest score)** |
| --- | --- | --- | --- | --- | --- |
| **Tuntland (2016)** | Multi-center study with n=225 participants who were followed for 10 weeks. After COPM interview additional questions to examine content validity and feasibility. Hypotheses testing with 4 predefined 'questions'. Comparison of identified occupations with filled categories in COPM (coverage, prioritization, relevance), and availability of scores. | Yes, items relevant for construct; frequency of prioritized occupations varied among the 9 occupational categories and there were sex- and age-specific variations. | Yes, only 7% of stated occupations could not be categorized, and 12% of patients reported items that could not be covered during the COPM interview, but almost all were categorized in retrospect by the PI (except for 'sleep and hearing', and 'to be independent'). | Yes, almost all participants (>99%) were able to define occupations, and to rate performance and satisfaction. Feasibility: About 10% of the participants described difficulties with answering questions and scoring, or regarded the instrument as less useful in the goal-setting process. | Doubtful  (analysis by 1 instead of 2 researchers) |

**Table S3.2.** Content validity, summary of literature review (n=1 study).
